# In vitro assessment of pacing as therapy for aortic regurgitation

**DOI:** 10.1136/openhrt-2018-000976

**Published:** 2019-05-24

**Authors:** Paolo Peruzzo, Francesca Maria Susin, Andrea Colli, Gaetano Burriesci

**Affiliations:** 1 Department of Civil, Environmental and Architectural Engineering, University of Padova, Padua, Italy; 2 Dipartimento di Scienze Cardiologiche Toraciche e Vascolari, Universita degli Studi di Padova, Padova, Italy; 3 UCL Mechanical Engineering, University College London, London, UK; 4 Ri.MED Foundation, Palermo, Italy

**Keywords:** pacemakers, ventricular fibrillation, heart failure treatment

## Abstract

**Background and objective:**

Clinical evaluation of pacing therapy in mitigating the aortic insufficiency after transchateter aortic valve implantation often gives contradictory outcomes. This study presents an in vitro investigation aimed at clarifying the effect of pacing on paravalvular leakage.

**Methods:**

A series of in vitro tests reproducing the heart operating changes clinically obtained by pacing was carried out in a 26 mm Edwards Sapien XT prosthesis with mild paravalvular leakage. The effect of pacing on the regurgitant volumes per cycle and per minute was quantified, and the energy and power consumed by the left ventricle were calculated.

**Results:**

Results indicate that though pacing results in some reduction in the total regurgitation per cycle, the volume of fluid regurgitating per minute increases substantially, causing overload of left ventricle.

**Conclusions:**

Our tests indicate no effective haemodynamic benefit from pacing, suggesting a prudential clinical use of this therapy for the treatment of postoperative aortic regurgitation.

**Key questions**

**What is already known about this subject?**

- Some in-vivo studies have debated the role of pacing to mitigate aortic regurgitation and its use as therapy to this aim.
- The discrepancies on the results are mainly due to some inaccuracies of in-vivo measurements and the improper use of aortic regurgitation index to assess the enhancement of the grade of insufficiency.

**What does this study add?**

- The study indicates that pacing is ineffective as a treatment for postoperative aortic regurgitation, and may result detrimental both in term of regurgitation and heart power demand.

**How might this impact on clinical practice?**

- The findings suggest a more widespread use of the left ventricular power as a heart performance parameter that allows an effective and consistent comparison between different heart beating conditions.

## Introduction

Significant postoperative aortic regurgitation due to paravalvular leakage (PVL) is a common complication after transcatheter aortic valve implantation (TAVI), with incidence ranging from 5% to 25%, associated with an increase in early and late postoperative mortality.^1–4^

Pacing has been adopted as temporary or permanent therapy to mitigate PVL in patients affected by moderate/severe regurgitation after TAVI.^5^ This approach is based on the concept that a shorter diastolic phase reduces the time available for blood to flow back into the ventricle, thus diminishing the ventricular overload.^6 7^ However, the clinical outcomes described in the literature are often contradictory.^7–11^ These inconsistencies can be attributed to the relatively reduced accuracy of in vivo measurements approaches,^12^ as well as the difficulty in comparing different courts of patients examined at various centres.

The present study aims at assessing in vitro the effect of pacing on the cardiac load and its impact on the power consumption of the heart. In order to allow direct comparison between the different operating conditions, an in vitro approach was adopted. This enables full control of the working parameters, as well as an accurate measurement of the functional quantities. Tests mimicking mild PVL after TAVI and different pacing were carried out, and the efficacy of pacing was verified by investigating its effect on the ventricular overload per beat and per unit time (ie, per minute). The distinction between these two time references becomes fundamental when adopting pacing, as quantities typically measured over a single heart cycle do not necessarily reflect the global benefit and may lead to erroneous interpretations of the results.

## Methods

**Figure 1:**
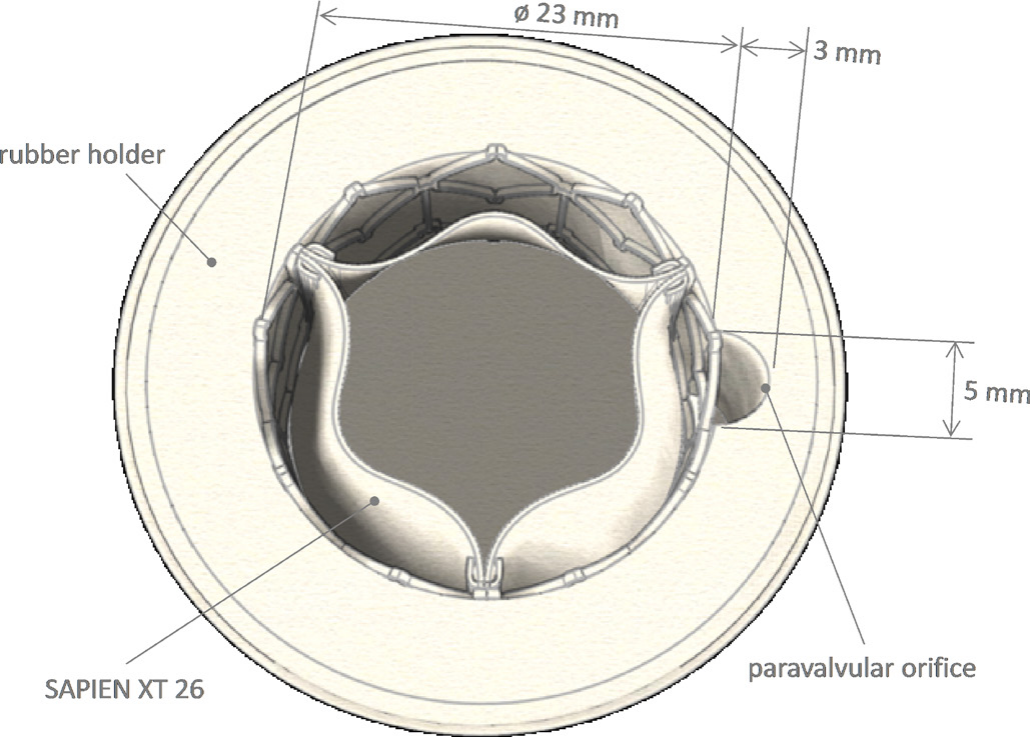
Sketch of the housing valve apparatus as reported by Burriesci *et al*.^13^

A 26 mm diameter Edwards SAPIEN XT was used as testing valve. This was released into a silicon cylindrical holder of 23 mm diameter by standard implantation procedure. The holder included a crescent shape axial grove (5 mm of minor axis, 3 mm of major semiaxis and total area A_orifice_ 12 mm^2^) (figure 1) causing mild PVL.^13^

Following well-developed methodologies for evaluation of cardiovascular devices and in vitro haemodynamics features,^14 15^ physiological pulsatile flow conditions were replicated in a hydromechanical pulse duplicator (ViVitro System, ViVitro Labs Inc, Victoria, Canada). The equipment includes a hydraulic circuit mimicking the human systemic circulation, comprising an atrial, a ventricular and an aortic chamber. The pulsatile flow is produced by a computer-driven piston pump, and localised dissipation and compliance chambers are placed downstream of the aortic root to account for the Windkessel effect. Flow through the ventricular and aortic chambers is measured with an electromagnetic flowmeter (model 501, Carolina Medical Electronics, Inc, King, North Carolina, USA), and pressure in the three chambers is recorded from three 8-French Millar microtip transducers (Millar Instruments, Houston, Texas, USA). Phosphate buffered saline solution at 37°C was adopted as testing fluid.

Six tests were carried out at different hydrodynamic working conditions, as reported in table 1. Heart rate (HR) was varied in the range 60–110 bpm. The cardiac output (CO) and mean aortic pressure (p_Ao_) were set to replicate conditions observed in vivo,^16 17^ but conveniently scaled to match the optimum operating range of the pulse duplicator. In particular, CO was increased about proportionally with HR in the range 3.4–6.2 L/min (ie, a range twice smaller than the one measured in vivo^14^), while p_Ao_ was maintained constant at 100 mm Hg. To better represent the changes in physiological contraction of the left ventricle with the increase of HR, the systolic percentage of the cycle was also extended, in agreement with the clinical evidence.^18^

**Table 1:** Summary of experimental work conditions and main measured parameters and postprocessing data

Work conditions				Measured parameters									
p_Ao_	HR	CO	Systole	SV	CV	LV	RV	RF	CFR	LFR	RFR	P	E
mm Hg	bpm	L/min	%	mL	mL	mL	mL	%	mL/min	mL/min	mL/min	W	J
	60	3.4	31.8	107.4 (0.05)	5.3 (0.6)	9.3 (2.0)	14.6 (2.5)	20.4 (3.5)	318 (4.6)	558 (15.5)	876 (19.3)	1.31 (0.02)	1.31 (0.02)
	70	4.0	31.9	104.6 (0.04)	5.5 (0.4)	8.9 (1.9)	14.4 (2.1)	20.4 (3.0)	385 (3.3)	623 (15.9)	1008 (17.6)	1.54 (0.02)	1.32 (0.01)
100	80	4.5	36.6	102.7 (0.05)	6.2 (0.6)	7.7 (1.6)	13.9 (1.7)	19.7 (2.4)	496 (5.4)	616 (14.3)	1112 (15.2)	1.80 (0.01)	1.35 (0.01)
	90	5.0	39.3	98.9 (0.05)	6.6 (0.8)	6.3 (2.0)	12.9 (2.5)	18.8 (3.6)	594 (7.6)	567 (19.0)	1161 (23.7)	1.99 (0.02)	1.33 (0.02)
	100	5.5	41.4	96.8 (0.03)	7.8 (0.9)	5.1 (1.8)	12.9 (2.2)	19.1 (3.2)	780 (9.0)	510 (18.0)	1290 (22.0)	2.16 (0.01)	1.29 (0.01)
	110	6.2	43.1	99.4 (0.00)	8.2 (1.7)	5.2 (1.9)	13.4 (2.3)	19.3 (3.4)	902 (17.8)	572 (19.9)	1474 (24.1)	2.60 (0.02)	1.42 (0.02)

Data are averaged over the 10 cycles; values in parentheses are the SD.

CFR, closing regurgitant mean flow rate; CO, cardiac output; CV, closing volume; HR, heart rate; LFR, Leakage mean flow rate; LV, leakage volume; RF, regurgitant fraction; RFR, Total regurgitant flow rate; RV, regurgitant volume; SV, stroke volume.

For each testing condition, the instantaneous pressures in the aortic and ventricular chambers and the flow rate thorough the valve were measured during the entire cycle, collecting 256 samples per cycle.

Data were averaged over 10 consecutive cycles and used to extract the following quantities:

- Stroke volume (SV (mL)).
- Closing regurgitant volume (CV (mL)), corresponding to the backflow as the valve leaflets move from the open to the closed configuration.
- Leakage volume (LV (mL)), corresponding to backflow occurring when the valve is fully closed.
- Total regurgitant volume (RV (mL)), equal to the sum of CV and LV.
- Regurgitant fraction (RF (%)), given by the ratio between RV and SV.
- Closing regurgitant mean flow rate (CFR (mL/min)), given by the product of CV and HR.
- Leakage mean flow rate (LFR (mL/min)), given by the product of LV and HR.
- Total regurgitant mean flow rate (RFR (mL/min)), given by the product of RV and HR.
- Energy per cycle (E (J)), calculated as ventricular pressure–volume integral (ie, ventricular pressure–volume loop area).
- Power (P (W)), calculated as the ratio between the energy E and the heart beat duration:
  (1). P=E⋅HR/60

## Results

The values determined for the above parameters at the different testing conditions are summarised in table 1.

**Figure 2:**
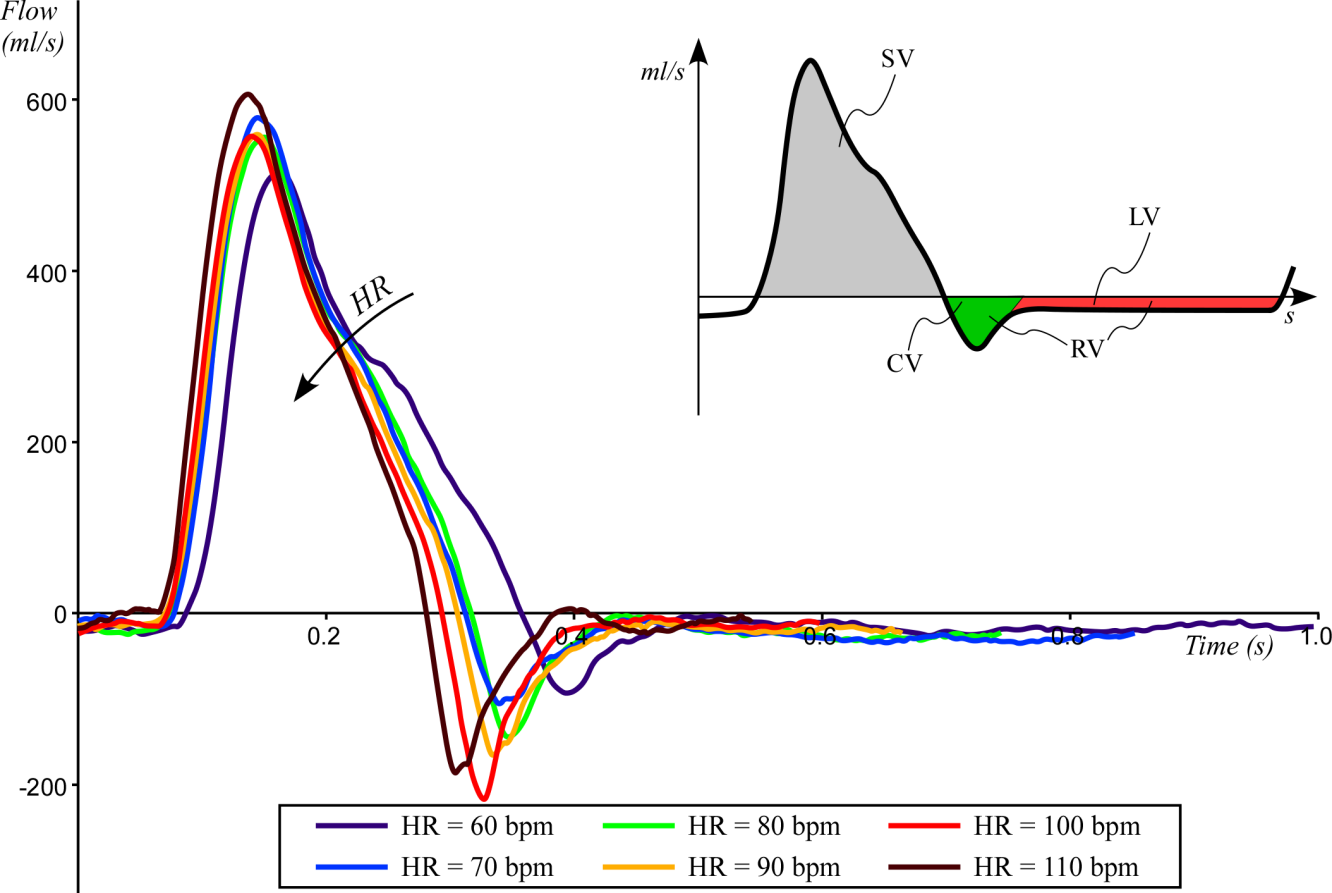
The averaged flow measured over the 10 cardiac cycles as function of the time. Flow was measured at HR=60 bpm (solid black line), HR=70 bpm (dashed black line), HR=80 bpm (dotted black line), HR=90 bpm (solid grey line), HR=100 bpm (dashed grey line) and HR=110 bpm (dotted grey line). CV, closing volume; HR, heart rate; LV, leakage volume; RV, regurgitant volume; SV, stroke volume.

The transaortic flow wave behaviour for the six study cases is depicted in figure 2. Peak flow ranges from 510 mL/s at HR=60 bpm to 610 mL/s at HR=110 bpm. Leakage volumetric flow rate (ie, closed valve condition) is similar for all conditions and ranges between 15 and 20 mL/s. On the contrary, regurgitant volumetric flow rate at peak increases from 90 mL/s at HR=60 bpm to 215 mL/s at HR=100 bpm.

**Figure 3:**
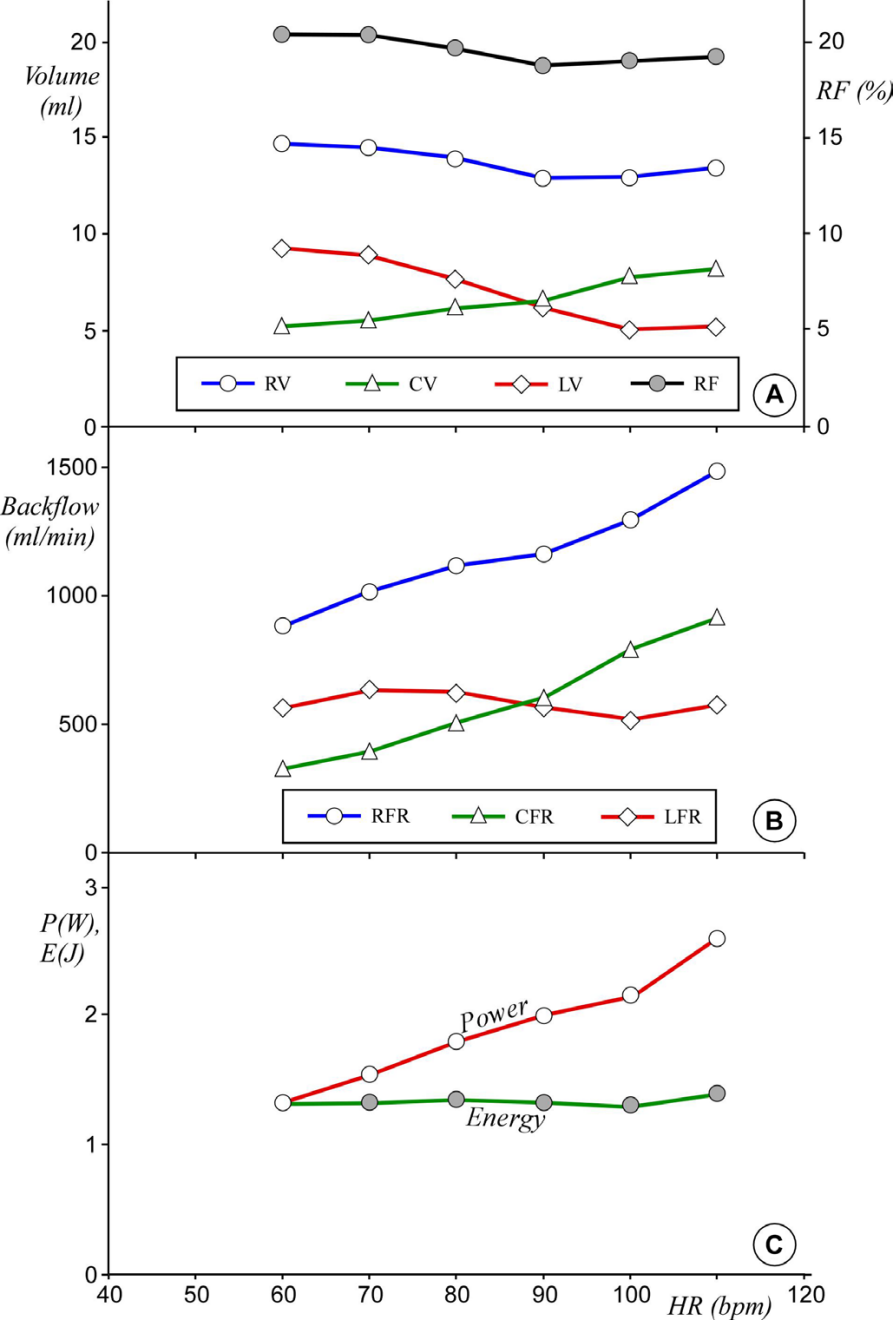
Measured parameter on aortic left ventricle performance as function of HR. (A) RV (white circle), the closing volume CV (triangle), the LV at closed valve LV (diamond) and along the second axis the RF (grey circle). Bars error are omitted for clarity. (B) Estimated total regurgitant mean flow rate (RFR) (circle) and the two contributions due to closing mean flow rate (CFR) (triangle) and leakage mean flow rate (LFR) (diamond) (bars error are negligible). (C) estimated power P (white circle) and energy per stroke E (grey circle) of the left ventricle (bars error are negligible). Data are averaged over the 10 cycles. CV, closing volume; HR, heart rate; LV, leakage volume; RF, regurgitant fraction; RV, regurgitant volume.

Figure 3 shows the main measured parameters, for example, backflow per cycle, mean backflow per minute, RF, energy loss and power per cycle, as described below.

The RV measured at the different testing configurations, with the contributions from both CV and LV, is summarised in the panel *a* of figure 3. As HR is raised from 60 bpm to 110 bpm, the closing volume increases from 5.3 mL to 8.2 mL; conversely, LV reduces from 9.3 mL to 5.1 mL. The RV keeps between 12.9 mL and 14.6 mL for all testing conditions. Similarly, the RF keeps about constant with HR, ranging from 20.4% (HR=60 bpm) to 18.8% (HR ≥90 bpm) (see figure 3A).

The RFR (ie, the total RV per minute) and the two contributions due to CFR and LFR are reported in panel B of figure 3. By raising HR from 60 bpm to 110 bpm, both regurgitant and closing mean flow rates increase monotonically from 875 mL/min to 1475 mL/min and from 320 mL/min to 900 mL/min, respectively, while leakage mean flow rate varies little, staying in the range between 510 mL/min and 623 mL/min.

Left ventricle energy per stroke E and power P as a function of HR are shown in the panel *c* of figure 3. E varies slightly between 1.3 and 1.4 J, while the associated power P doubles from 1.3 to 2.6 W.

## Discussion

Various studies performed in the 1960s with the dye dilution technique observed a reduction of RV in patients with increased^8^ HR and in patients with severe RF.^9^ Conversely, similar measurements with electromagnetic velocimetry performed by Brawley and Morrow^10^ in the same period did not produce consistent changes in RV with higher HR. This was confirmed by Judge *et al* 11 who, in the early 1970 s, reported that though the increase of HR induces a rise in CO and a reduction of SV and RF, it has no significant effect on the RV per minute. In a later study from Firth *et al*,^7^ the survey of 12 patients indicated that though increased HRs were associated with large reductions in RV per stroke, only minor reductions of RF could be observed and no substantial modification in the RV per minute. Again, these conclusions are disputed by a recent study by Ali *et al* 5 that reports clinical benefits in patients affected by aortic regurgitation after treatment with permanent pacing. The efficacy of the therapy is assessed by an overall enhancement of the aortic regurgitation index (ARI), that is, the ratio of the difference between the diastolic blood pressure and the left ventricular end-diastolic pressure (LVEDP) to the systolic blood pressure (ARI=(DBP−LVEDP)/SB).^19^

Some of the discrepancies in these studies may be justified by the use of different in vivo measurement techniques, as well as the limited accuracy of clinically available anemometric methodologies. This limitation is acknowledged in a topical work by Pibarot *et al*, which demonstrates the relatively low resolution of in vivo measurements through transoesophageal echo (the best cost–benefit ratio methodology for in vivo flow measurements) in quantifying the RV, and questions the reliability of ARI, especially when it is used to grade regurgitation as a function of HR.^12^

In the present study, for the first time, in vitro tests are carried out in order to verify and quantify the effect of pacing in presence of mild aortic regurgitation after TAVI. Flow measurement shows that the leakage rate determined in the diastolic period after full valve closure (LV per unit time) does not vary substantially for the tested conditions (between 15 mL/s and 20 mL/s), and it is consistent in value with the leakage rate expected from fluid dynamics theory (for more detail, see online supplementary appendix A). This is not surprising, because the instantaneous diastolic regurgitant flow rate is strictly dependent on the mean diastolic pressure difference (Δp_D_) (ie, the differential pressure acting across the valve in diastole) that, due to the choice of setting a fixed value for the mean aortic pressure (100 mm Hg), is similar for all testing cases. This implies that, as the diastolic period reduces with pacing, LV over a single cardiac cycle decreases about proportionally, as reflected in the diagram in figure 3A. On the contrary, the variation of CV and LV are counterbalanced, so that the total RV experiences only a small reduction of about 10% with increased HR. The increment of closing volume with HR observed in vitro is determinant to maintain the RV constant. No reference was found in the clinical literature to support or confute the rise in closing volume with pacing; however, even assuming a constant value of closing volume for all tests (eg, CV=5 mL) the reduction of RF would be at most 5% and hence insufficient to mitigate effectively the valve insufficiency. Similarly to RV, also the effect of pacing on the RF is reduced, as shown in figure 3A.

10.1136/openhrt-2018-000976.supp1 Supplementary data

A comprehensive analysis of the phenomenon has to take into account the variation in the number of cycles per minute resulting from pacing. Conversion of the regurgitant parameters into mean flow rates modifies the diagram in figure 3A as shown in figure 3B. Trivially, the global volume of blood returning back from the aorta into the ventricle per each minute is given by the RV occurring at each cycle, multiplied by the number of cycles in 1 min. Hence, all quantities defining the backflow increase proportionally with HR. It is interesting observing that the conversion from LV (defined per cycle) to leakage mean flow rate (defined per minute) results into a stabilisation of the latter, which becomes approximately constant. This is justified by the fact that the instantaneous diastolic regurgitant flow rate is about constant, as previously discussed, and the total diastolic period decreases only slightly with HR rising from 60 to 110 (in a minute, the cumulative time of all diastoles varies from about 41 s at 60 bpm to about 34 s at 110 bpm).

The most critical finding of the study is that the overall effect of pacing is a significant increment of the regurgitant mean flow rate, necessarily associated with a higher overload for the heart.

Such result is well reflected in the behaviour of the energy consumed by the left ventricle during the single cycle, E, and of the power, P, corresponding to the energy consumption per minute. E is almost constant for all HRs, but the overall work expended per minute increases, as shown in the diagram of the required ventricular power P, which doubles within the tested HR range (see figure 3C).

The present analysis is limited to the case of mild prosthetic aortic valve regurgitation, a condition that is common in patients after transcatheter aortic valve replacement (TAVR), as well as the need for post implantation pacing to overcome the development of complete AV block as recently reported in multicentre studies, in which the permanent pacemaker was implanted up to 17.4% of the patients.^20 21^ However, the outcomes can be confidently extended also in presence of a moderate regurgitation, as well as a severe grade of PVL, although, in the latter, reoperation is still the only indicated therapeutic option, thus limiting the number of patient requiring pacing.

Of course, the presented study is based on a number of necessary assumptions that may affect the relevance of the results, such as the adoption of idealised anatomical and operating conditions. In particular, the use of a rigid aortic root and a straight paravalvular orifice of constant semielliptical cross section is a generalisation, which suggests some caution when transposing the presented results to the clinical case. Also, the fluid behaviour is necessarily dependent on the dynamic of the selected valve, and on the features imposed to describe the contraction phase (in the present in vitro case, the imposed contraction of the ventricular chamber).

The increment in mean aortic pressure typically observed with increased HR^17^ is expected to result into larger RVs than the volumes measured here, with constant p_Ao_.

Another approximation consists in the use of saline solution, which might result into different velocity profiles inside the paravalvular orifice, due to the reduced viscosity (this is about one fourth as the viscosity of blood at 37°C). However, theoretical considerations indicate that, for the used working conditions, the effect of viscosity is expected to be negligible.^22^

## Conclusions

This study presents, for the first time, a systematic study of the efficacy of pacing as a treatment for PVL. The left ventricular operating conditions under pacing were simulated in vitro on a Sapien XT of size 26 mm, imposing mild paravalvular leakage.

The RVs per cycle and per minute, the energy and power consumed by the ventricle in a single beat were estimated at different HRs.

Although the shorter diastolic phase for high HRs causes a slight reduction of RF measured over the cardiac cycle, the volume of fluid regurgitating per minute increases substantially, causing overload of the left ventricle.

The presented results indicate that pacing produces no effective haemodynamic benefits in patients with AR, in terms of regurgitation, and can result in significant increases in heart power demand. Moreover, collateral complications may arise due a persistent non-physiological shear stress, whose quantification has been demonstrated being rather complex.^23^ This suggests a prudential use of temporary or permanent therapy for the treatment of postoperative aortic regurgitation.

The study demonstrate that parameters typically adopted to evaluate and quantify aortic insufficiency (eg, RV and RF), based on a single cardiac cycle, are not adequate to capture the effect of HR on the cardiac performance. This may result in erroneous interpretation and quantification of the clinical benefit deriving from pacing, leading to its improper adoption.

To correctly evaluate the efficacy of this therapy, alternative parameters based on a fixed chronological period including several heart beats (eg, 1 min) should be used. In particular, we recommend the use of the left ventricular power as a heart performance parameter, as it allows an effective and consistent comparison between operating conditions characterised by different heart rhythms. In addition, other methodologies might be explored by considering coexisting features related to altered aortic valve haemodynamics, such as the sound of regurgitant jet.^24–26^

Finally, further specific studies would be appropriate to frame the role of the energy and power consumption in the perspective of optimisation of heart performance. This aspect may potentially enhance also the comprehension of the cardiac physiopathology characterised by ventricular remodelling.

## References

[1] Gurvitch R, Wood DA, Tay EL, et al Transcatheter aortic valve implantation: durability of clinical and hemodynamic outcomes beyond 3 years in a large patient cohort. Circulation 2010;122:1319–27. 10.1161/CIRCULATIONAHA.110.948877 20837893

[2] Tamburino C, Capodanno D, Ramondo A, et al Incidence and predictors of early and late mortality after transcatheter aortic valve implantation in 663 patients with severe aortic stenosis. Circulation 2011;123:299–308. 10.1161/CIRCULATIONAHA.110.946533 21220731

[3] Moat NE, Ludman P, de Belder MA, et al Long-term outcomes after transcatheter aortic valve implantation in high-risk patients with severe aortic stenosis: the U.K. TAVI (United Kingdom transcatheter aortic valve implantation) registry. J Am Coll Cardiol 2011;58:2130–8. 10.1016/j.jacc.2011.08.050 22019110

[4] Gotzmann M, Pljakic A, Bojara W, et al Transcatheter aortic valve implantation in patients with severe symptomatic aortic valve stenosis-predictors of mortality and poor treatment response. Am Heart J 2011;162:238–45. 10.1016/j.ahj.2011.05.011 21835283

[5] Ali O, Salinger MH, Levisay JP, et al High pacing rates for management of aortic insufficiency after balloon aortic valvuloplasty or transcatheter aortic valve replacement. Cathet Cardiovasc Intervent 2014;83:162–8. 10.1002/ccd.24902 23441087

[6] Corrigan DJ On permanent patency of the mouth of the aorta, or inadequacy of the aortic valves. Edinburgh Med Surg J 1832;37:225–45. PMC5818233 30331470

[7] Firth BG, Dehmer GJ, Nicod P, et al Effect of increasing heart rate in patients with aortic regurgitation. Effect of incremental atrial pacing on scintigraphic, hemodynamic and Thermodilution measurements. Am J Cardiol 1982;49:1860–7. 6282104 10.1016/0002-9149(82)90203-x

[8] Warner HR, Toronto AF Effect of heart rate on aortic insufficiency as measured by a dye-dilution technique. Circ Res 1961;9:413–7. 10.1161/01.RES.9.2.413 13783191

[9] Rothlin M, Rutishauser W, Wirz P Role of heart rate on the hemodynamics of aortic incompetence. Z Kreislaufforsch 1968;57.

[10] Brawley RK, Morrow AG Direct determinations of aortic blood flow in patients with aortic regurgitation. Effects of alterations in heart rate, increased ventricular preload or afterload, and isoproterenol. Circulation 1967;35:32–45. 6016054 10.1161/01.cir.35.1.32

[11] Judge TP, Kennedy JW, Bennett LJ, et al Quantitative hemodynamic effects of heart rate in aortic regurgitation. Circulation 1971;44:355–67. 10.1161/01.CIR.44.3.355 5097439

[12] Pibarot P, Hahn RT, Weissman NJ, et al Assessment of paravalvular regurgitation following TAVR: a proposal of unifying grading scheme. JACC Cardiovasc Imaging 2015;8:340–60. 10.1016/j.jcmg.2015.01.008 25772838

[13] Burriesci G, Peruzzo P, Susin FM, et al In vitro hemodynamic testing of Amplatzer plugs for paravalvular leak occlusion after transcatheter aortic valve implantation. Int J Cardiol 2016;203:1093–9. 10.1016/j.ijcard.2015.11.106 26642371

[14] Toninato R, Salmon J, Susin FM, et al Physiological vortices in the sinuses of Valsalva: an in vitro approach for bio-prosthetic valves. J Biomech 2016;49:2635–43. 10.1016/j.jbiomech.2016.05.027 27282961 PMC5061069

[15] Colli A, Besola L, Bizzotto E, et al Edge-to-edge mitral valve repair with transapical neochord implantation. J Thorac Cardiovasc Surg 2018;4:1–6. 10.1016/j.jtcvs.2018.02.008 29510937

[16] Rodeheffer RJ, Gerstenblith G, Becker LC, et al Exercise cardiac output is maintained with advancing age in healthy human subjects: cardiac dilatation and increased stroke volume compensate for a diminished heart rate. Circulation 1984;69:203–13. 10.1161/01.CIR.69.2.203 6690093

[17] Wilkinson IB, MacCallum H, Flint L, et al The influence of heart rate on augmentation index and central arterial pressure in humans. J Physiol 2000;525:263–70. 10.1111/j.1469-7793.2000.t01-1-00263.x 10811742 PMC2269933

[18] Bombardini T, Sicari R, Bianchini E, et al Abnormal shortened diastolic time length at increasing heart rates in patients with abnormal exercise-induced increase in pulmonary artery pressure. Cardiovasc Ultrasound 2011;9 10.1186/1476-7120-9-36 PMC3268730 22104611

[19] Sinning J-M, Hammerstingl C, Vasa-Nicotera M, et al Aortic regurgitation index defines severity of peri-prosthetic regurgitation and predicts outcome in patients after transcatheter aortic valve implantation. J Am Coll Cardiol 2012;59:1134–41. 10.1016/j.jacc.2011.11.048 22440213

[20] Popma JJ, Deeb GM, Yakubov SJ, et al Transcatheter aortic-valve replacement with a self-expanding valve in low-risk patients. N Engl J Med 2019;0. 10.1056/NEJMoa1816885 30883053

[21] Mack MJ, Leon MB, Thourani VH, et al Transcatheter aortic-valve replacement with a Balloon-Expandable valve in low-risk patients. N Engl J Med 2019;0. 10.1056/NEJMoa1814052 30883058

[22] Sherman M A power-law formulation of laminar flow in short pipes. J Fluids Eng 1992;114:601–5. 10.1115/1.2910073

[23] Toninato R, Fadda G, Susin FM A red blood cell model to estimate the hemolysis fingerprint of cardiovascular devices. Artif Organs 2018;42:58–67. 10.1111/aor.12937 28722138

[24] Susin FM, Tarzia V, Bottio T, et al In-vitro detection of thrombotic formation on bileaflet mechanical heart valves. J Hear Valve Dis 2011;20:378–86. 21863649

[25] Romata C, Susin FM, Cambi A, et al Comparative classification of thrombotic formations on bileaflet mechanical heart valves by phonographic analysis. J Artif Organs 2011;14:100–11. 10.1007/s10047-011-0562-z 21448607

[26] Melan G, Bellato A, Susin FM, et al Ultrasound phonocardiography for detecting thrombotic formations on bileaflet mechanical heart valves. J Heart Valve Dis 2013;22:828–36. 24597405

