# Supplementary material for: In vitro assessment of pacing as therapy for aortic regurgitation
Source: Open Heart. 2019 May 24;6(1):e000976. doi: 10.1136/openhrt-2018-000976 (PMC6546189; doi:10.1136/openhrt-2018-000976)
Supplement: Supplementary data [file openhrt-2018-000976supp001.pdf]

APPENDIX A

The mean diastolic pressure difference measured between the ventricular and aortic chamber (ΔP_D) is large enough to consider the length of the orifice insignificant; i.e. the leakage flow only depends on chambers pressures and fluid density (ρ)[22], which is similar for saline solution and blood. In this condition the leakage rate can be estimated by the conservation of the fluid energy per unit of volume between the aortic root and the left ventricle. With reference to the notation in Fig. 4, the energy balance reads:

$$p_{Ao} = p_V + \frac{\rho}{2}U^2; \quad \text{(A1)}$$

where p_{Ao} and p_V are the mean aortic and the mean ventricle pressure, respectively, and U is the velocity of the leaking jet. Being the mean diastolic pressure difference equal to the difference between aortic and ventricular pressure ($\Delta P_D = p_{Ao} - p_V$), the velocity U_{leak} can be expressed as:

$$U = \sqrt{2 \cdot \Delta p_D / \rho} \quad \text{(A2)}$$

Since in the presented in-vitro tests ΔP_D is about 100 mmHg, Eq. (A2) gives a value of U = 1.6 m/s.

The leakage flow rate (Q) is given by the product of the jet velocity and and the area of its cross section, i.e.:

$$Q = U \cdot A_{jet} = U \cdot A_{orifice} \cdot c_c; \quad \text{(A3)}$$

where c_c is the contraction coefficient which accounts for the effective orifice area of the section, and is equal to 0.61. The rough estimation of the flow rate given by the Eq. (A3) ($A_{orifice}$ is assumed to be circular) is Q = 12 ml/s. This is not far from the 15-20 ml/s measured in the end diastolic period (see Fig. 2), which also consider a not negligible fraction due to diffusive paravalvular leakage, distributed along the contact region between the frame and the host housing.

Peruzzo P, *et al. Open Heart* 2020; 6:e000976. doi: 10.1136/openhrt-2018-000976
